# Electrodeposited Ni-Rich Ni–Pt Mesoporous Nanowires for Selective and Efficient Formic Acid-Assisted Hydrogenation of Levulinic Acid to γ-Valerolactone

**DOI:** 10.1021/acs.langmuir.1c00461

**Published:** 2021-04-07

**Authors:** Albert Serrà, Raül Artal, Laetitia Philippe, Elvira Gómez

**Affiliations:** † Laboratory for Mechanics of Materials and Nanostructures, Empa, Swiss Federal Laboratories for Materials Science and Technology, Feuerwerkerstrasse 39, CH-3602 Thun, Switzerland; ‡ Grup d’Electrodeposició de Capes Primes i Nanoestructures (GE-CPN), Departament de Ciència de Materials i Química Física, Universitat de Barcelona, Martí i Franquès, 1, E-08028 Barcelona, Catalonia, Spain; § Institute of Nanoscience and Nanotechnology (IN^2^UB), Universitat de Barcelona, E-08028 Barcelona, Catalonia, Spain

## Abstract

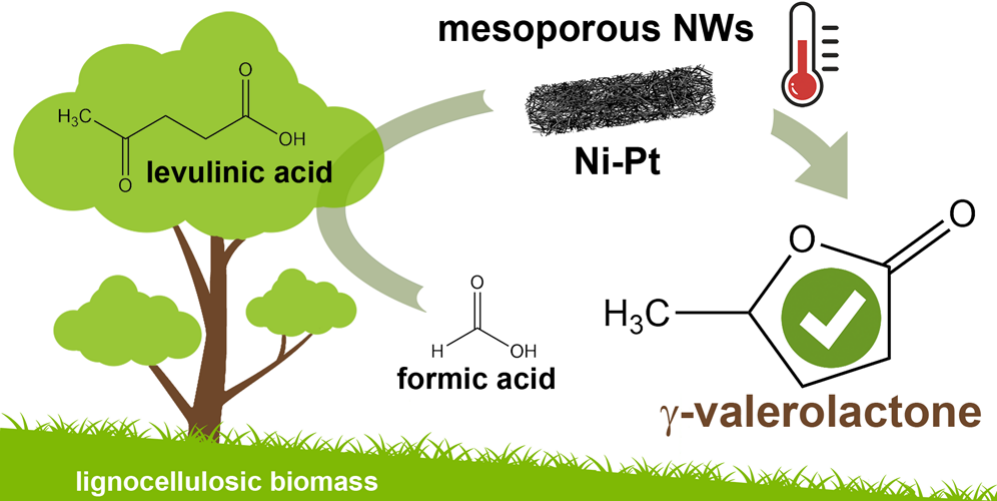

In pursuit of friendlier conditions for the preparation of high-value biochemicals, we developed catalytic synthesis of γ-valerolactone by levulinic acid hydrogenation with formic acid as the hydrogen source. Both levulinic and formic acid are intermediate products in the biomass transformation processes. The objective of the work is twofold: the development of a novel approach for milder synthesis conditions to produce γ-valerolactone and the reduction of the economic cost of the catalyst. Ni-rich Ni–Pt mesoporous nanowires were synthesized in an aqueous medium using a combined hard–soft-template-assisted electrodeposition method, in which porous polycarbonate membranes controlled the shape and the Pluronic P-123 copolymer served as the porogen agent. The electrodeposition conditions selected favored nickel deposition and generated nanowires with nickel percentages above 75 atom %. The increase in deposition potential favored nickel deposition. However, it was detrimental for the porous diameter because the mesoporous structure is promoted by the presence of the platinum-rich micelles near the substrate, which is not favored at more negative potentials. The prepared catalysts promoted the complete transformation to γ-valerolactone in a yield of around 99% and proceeded with the absence of byproducts. The coupling temperature and reaction time were optimized considering the energy cost. The threshold operational temperature was established at 140 °C, at which, 120 min was sufficient for attaining the complete transformation. Working temperatures below 140 °C rendered the reaction completion difficult. The Ni_78_Pt_22_ nanowires exhibited excellent reusability, with minimal nickel leaching into the reaction mixture, whereas those with higher nickel contents showed corrosion.

## Introduction

The exponential growth in the concentration of atmospheric carbon dioxide owing to the overdependence on fossil fuels for meeting energy requirements is causing global warming at alarming levels. The exploration of renewable energy sources is an important solution for addressing these challenges. Lignocellulosic wastes have been identified as vital sources of energy for the development of a sustainable economy. These biomasses are the only renewable raw materials with the unique ability to produce organic molecules. The transformations of lignocellulosic biomass can produce several renewable platform molecules, such as 5-hydroxymethylfurfural (5-HMF) and levulinic acid (LA), and these transformations have been thoroughly investigated.^1−3^ Thanks to its reactive keto and carboxylic acid functional groups, the strategic transformation of LA into further value-added biofuels and biochemicals is possible, and several valuable biochemicals such as fuel additives, fragrances, solvents, pharmaceuticals, and plasticizers have been synthesized using such transformations.^4,5^ Among these high-value chemicals, γ-valerolactone (GVL) has received considerable attention as a raw material for the synthesis of valuable biochemicals, such as food additives, drug intermediates, and novel biofuels. GVL is a key LA derivative and is synthesized by the hydrogenation of LA.^6−8^

The hydrogenation of LA to GVL has been performed in gaseous or liquid phases under homogeneous or heterogeneous catalysis at 60–270 °C, using high H_2_ pressures (30–150 bar). The high energy consumption required to vaporize LA makes this approach less attractive compared to the liquid-phase hydrogenation. Typically, the conversion of LA to GVL can be performed using three different hydrogen sources: (i) molecular hydrogen from an external source, (ii) hydrogen generated in situ from the decomposition of formic acid (FA), or (iii) by the Meerwein–Ponndorf–Verley reaction using alcohols.^9−11^ Further, to exploit the facile separation of the liquid products upon hydrogenation, the conversion of LA to GVL has been extensively studied using molecular hydrogen under heterogeneous catalysis.^12−17^

**Scheme 1:**
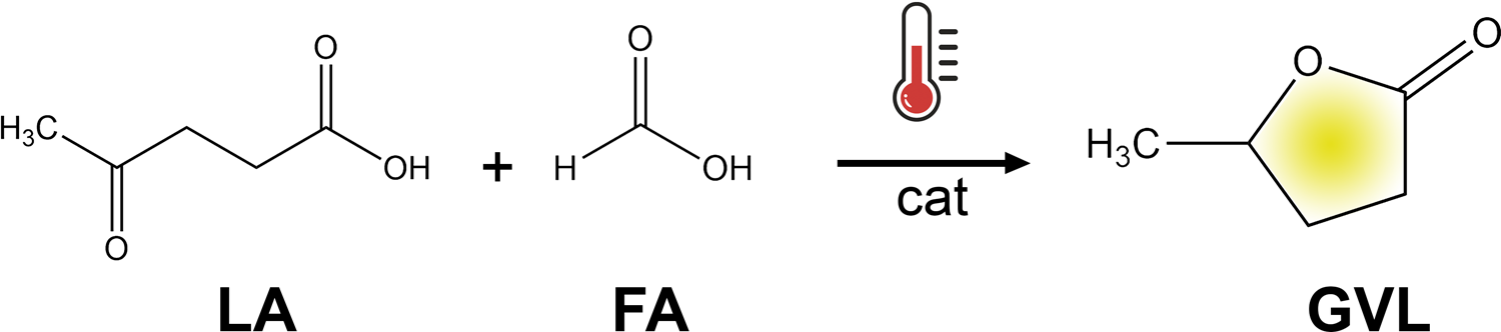
Conversion of LA and FA to γ-Valerolactone over Various Heterogeneous Catalysts under mild conditions

Biomass is converted into LA and FA by the acid-catalyzed hydrolysis of various biomass components. For example, cellulose is converted into LA and FA in the presence of dilute mineral acids such as hydrochloric and sulfuric acids. Biomass is initially broken into low-molecular-weight fragments and ultimately to glucose, which then decomposes initially into 5-HMF and then into LA and FA.^18−21^ Importantly, the equimolar mixture of LA and FA can be efficiently converted into γ-valerolactone over various heterogeneous catalysts under mild conditions (Scheme 1). The use of FA as the hydrogen source reduces the operational risk associated with the direct use of hydrogen gas. Also, it allows a more sustainable and cleaner production of GVL by preventing waste generation because FA is also obtained during the production of LA from biomass.^18,22−25^

Precious metal catalysis of LA hydrogenation has been widely investigated owing to its superior performance in the reaction. However, these metals are costly due to their low natural abundance. In this context, the use of bimetallic catalysts of low-cost transition metals for obtaining bimetallic alloys with promising LA hydrogenation reactivity is important considering their natural abundance and economic sustainability. Nickel is a low-cost metal used industrially for hydrogenation, methanation, and steam reforming reactions. Due to its hydrogenation ability, several Ni-based catalysts have been tested in the reduction of LA.^26,27^

The use of porous materials as catalysts is an attractive solution for minimizing the volume of high-cost materials, and particularly, mesoporous materials offer high surface-to-volume ratios and provide more reaction sites than their traditional bulk counterparts.^28−33^ Electrodeposition is one of the most promising technologies for the preparation of mesoporous catalysts due to the low preparation time, low cost, and the straightforward experimental setup.^34−36^ Electrochemistry is a versatile tool for synthesizing mesoporous micro- and nanoarchitectured catalysts via templated electrodeposition using hard and/or soft templates, if not both simultaneously, or via electrochemical dealloying.^37−39^ During the last two decades, the relatively high level of control of pore-size distribution and pore definition added to the low cost, high-scalability potential, and ease of operation of soft-templated electrodeposition have been immensely significant drivers of electrodeposition for mesoporous fabrication.^34,40−44^ Among the different soft-template systems, block copolymer-templated electrodeposition offers greater robustness and uniform controllable mesoporosity.^45,46^ Additionally, the use of electrochemical methods such as electrodeposition allows their simultaneous use as both soft and hard templates, which affords control of both the shape and porosity of the prepared material.^34,40−44^ Notably, the combination of hard- and soft-template-assisted electrodeposition provides a huge specific surface in the prepared Ni–Pt structures. The block copolymer and applied potential are responsible for the mesoporous character and the Ni/Pt ratio, respectively. In this work, new mesoporous Ni-rich Ni–Pt nanowires (NWs) have been prepared and tested as heterogeneous catalysts for the hydrogenation of LA to GVL using formic acid as the sole hydrogen source.

## Experimental Section

### Synthesis of Mesoporous Ni–Pt Catalysts

#### Electrochemical Media

The micellar solution consisted of an aqueous solution of 3 mM Na_2_PtCl_6_, 200 mM NiCl_2_, 200 mM H_3_BO_3_, 25 mM NH_4_Cl, and 10 g L^–1^ block copolymer poly(ethylene glycol)-*b*-poly(propylene glycol)-*b*-poly(ethylene glycol), also known as Pluronic P-123 (P-123) and abbreviated as PEG-*b*-PPG-*b*-PEG. The pH was adjusted to 2.7 with a 1 M HCl solution. All chemicals were purchased from Sigma-Aldrich. Solutions were prepared with Milli-Q water (Millipore) with a resistivity of 18.2 MΩ cm.

#### Electrodeposition and Characterization of Mesoporous Films and Nanowires

The setup for the electrochemical preparation and characterization of the mesoporous structures involved an Autolab PGSTAT30 potentiostat–galvanostat controlled by GPES (version 4.9) software using a three-electrode configuration. Pt spiral and Ag|AgCl|KCl (3 M) were used as counter and reference electrodes, respectively. Si/Ti (15 nm)/Au (100 nm) substrates or commercial polycarbonate (PC) membranes (nominal pore size = 100 nm), covered with a 100 nm gold layer on one side, were used as working electrodes for electrosynthesizing films or nanowires (NW), respectively. Prior to deposition, the polycarbonate membranes were weighed to determine the mass of the deposited NWs. The electrochemical medium, deaerated by argon bubbling before each experiment, was maintained in an argon atmosphere during the experiments. All measurements were performed at 30.0 ± 0.1 °C in stagnant conditions. Films were washed with tetrahydrofuran (THF) at 40.0 ± 0.1 °C for 30 s and rinsed exhaustively with Milli-Q water and were subjected to 100 W of O_2_ plasma to remove residual polymers. The electrodeposited NWs were dried and weighed several times until a constant weight was attained that would allow determining the total mass of synthesized NWs. Next, the Au layer was removed by etching the Au using a saturated solution of I_2_/I^–^. The polycarbonate membrane was dissolved with chloroform and the released NWs were cleaned 10 times with chloroform, followed by washing five times with ethanol and five times with water under ultrasound stirring.

Field-emission scanning electron microscopy (FE-SEM, Jeol JSM 7100 F, Hitachi S-4800, and Hitachi H-4100FE), equipped with energy-dispersive X-ray spectroscopy detectors was used to characterize the morphology, architecture, and elemental composition of mesoporous films and NWs. Elemental composition was also confirmed using X-ray fluorescence (XRF) spectrometry with a Fischerscope X-ray XDV-SDD. High-resolution transmission electron microscopy (HR-TEM, Jeol 2100) was also used to visualize the morphology of mesoporous NWs. X-ray photoelectron spectroscopy (XPS, PHI ESCA-5500 MultiTechnique system (Physical Electronics), base pressure = 5 × 10^–10^ mbar, excitation source = monochromatic Al Kα radiation (i.e., Al Kα line of 1486.6 eV energy and 350 W)) was used to further investigate the chemical composition and the chemical state of the elements in the prepared deposits. X-ray diffraction (XRD, Bruker D8 Discovery diffractometer in the Bragg–Brentano configuration with Cu Kα radiation) was also employed to determine the crystal phase of deposits. The N_2_ adsorption–desorption isotherms at 77 K (Tristar-II device (Micromeritics)) were determined to measure the Brunauer–Emmett–Teller (BET) surface areas of deposited materials.

### Levulinic Acid Hydrogenation Tests

The hydrogenation of LA to GVL was performed in a 10 mL autoclave (autogenic conditions). In a standard LA hydrogenation test, 5 mg of NWs and 4 mL of a solution of FA (496 mg) and LA (1 g) were maintained at a fixed temperature range of 120–180 °C for 45–180 min. The zero time was recorded when the temperature equaled the reaction temperature. After the reaction, the autoclave was cooled to room temperature, and the catalyst was collected by magnetic field recollection and/or filtration. Product identification, conversion, and yields of reaction were investigated via high-performance liquid chromatography (HPLC), gas chromatography coupled with a mass spectrometer (GC-MS), and ^1^H nuclear magnetic resonance (NMR) spectroscopy. The reaction mixture was analyzed by HPLC using an Agilent 1260 Infinity with a C18 column (ZORBAX Eclipse XDB, 4.6 mm of internal diameter × 250 mm, 5 μm packing). The elution phase, consisting of 10% (v/v) acetonitrile and 90% (v/v) of 0.1% orthophosphoric acid, was performed at a wavelength of 276 nm, with a flow rate of 500 μL min^–1^ and a constant column temperature of 35 °C. The yields of the products were analyzed by GC-MS with a Shimadzu GC-MS-QP 2020 equipped with a wax capillary column (30 m in length, 0.25 mm in internal diameter, and 0.25 μm in film thickness). The column temperature, initially kept at 50 °C for 4 min, was increased to 250 °C at a ramping rate of 20 °C min^–1^ and maintained for 10 min using helium as the carrier gas. ^1^H NMR (400 MHz) spectra were recorded with a Varian Mercury spectrometer (Varian Inc.) and processed with Mestrelab’s Mnova software (version 10.0). The turnover frequency (TOF), representing the moles of the reactant converted per hour per mole of Ni–Pt on the surface of the catalyst, was estimated

## Results and Discussion

### Electrochemical Characterization

**Figure 1:**
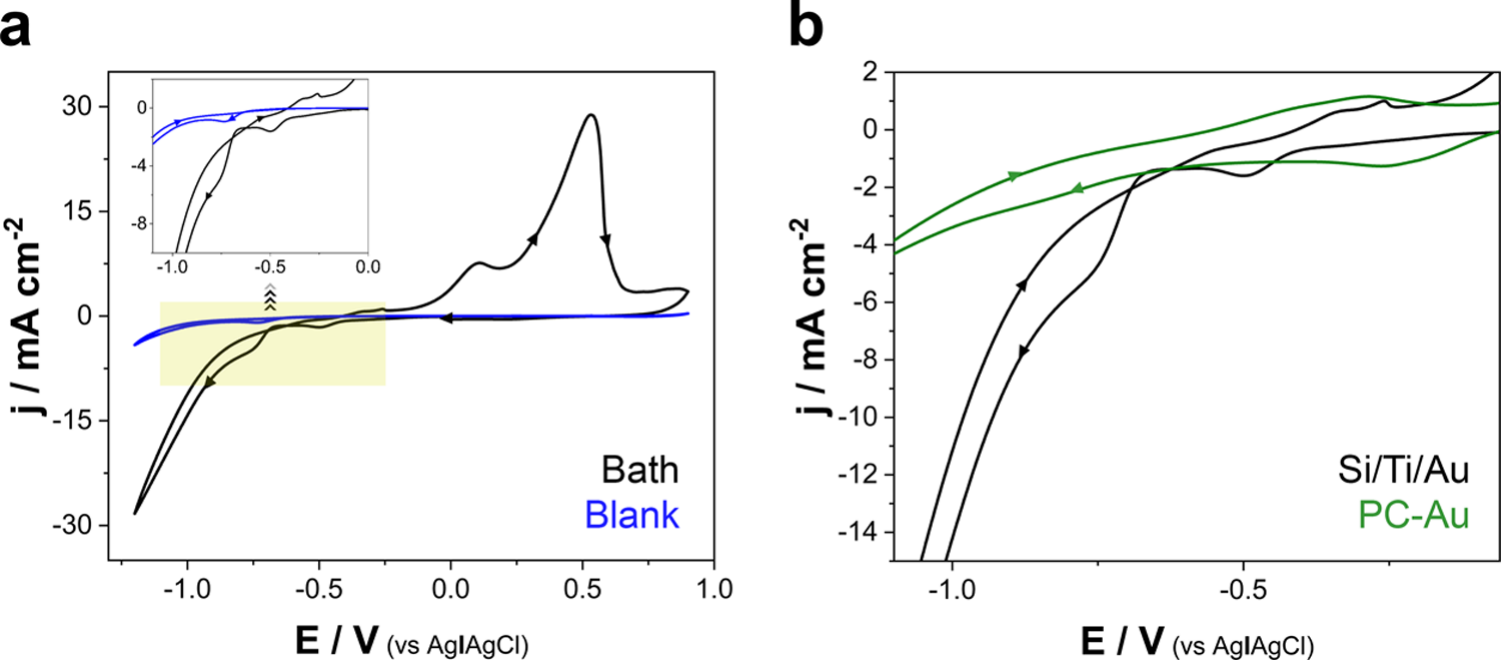
(a) CVs of 3 mM Na_2_PtCl_6_ + 200 mM NiCl_2_ + 200 mM H_3_BO_3_, 25 mM NH_4_Cl + 10 g L^–1^ P-123 solution (black line) and blank solution without metallic precursors (blue line) on Si/Ti/Au at 50 mV s^–1^ and 30.0 °C. (b) Reduction region of the CVs of the Ni–Pt bath on Si/Ti/Au (black line) and the polycarbonate membrane coated with an Au layer (green line) at 50 mV s^–1^ and 30.0 °C.

Cyclic voltammetries (CVs) were recorded at a scan rate of 50 mV s^–1^ under stagnant conditions to establish the optimal electrodeposition conditions for the electrosynthesis of mesoporous Ni-rich Ni–Pt NWs. In Figure 1a, the black line shows the CV of Ni–Pt deposition on Si/Ti/Au, whereas the blue line corresponds to the blank solution without the metallic precursors (i.e., NH_4_Cl + H_3_BO_3_ + P-123). The voltammetric profile corresponding to the electrodeposition process displayed a current associated with the Pt-reduction current at around −0.3 V vs Ag|AgCl|Cl^–^, followed first mainly by the reduction of protons on the previously deposited Pt around −0.8 V vs Ag|AgCl|Cl^–^, and practically, indicated simultaneous Ni–Pt alloy deposition.^47−52^ The large deposition current at the more negative potentials is attributable to the simultaneous codeposition of Ni and Pt as well as to the hydrogen coevolution. The current density in the codeposition zone increased with the increase in the Ni(II) concentration in the electrochemical bath, thus confirming the codeposition of Ni. More negative potentials were not considered, however, due to the significant level of hydrogen coevolution.^47−50^ A reduction of protons was also detected in the blank solution, albeit at a less significant level than when Pt and Ni–Pt were deposited, owing to the low catalytic power directed toward the reaction with the Au substrate. The weak peak at approximately −0.8 V vs Ag|AgCl|Cl^–^ in the blank solution was highly sensitive to the solution’s pH (i.e., proton concentration), which confirmed that the process corresponds to proton reduction. In the reverse scan, although no oxidation current was observed for the blank solution, whereas three separate peaks were observed for the Ni–Pt baths. The primary oxidation peak, appearing at approximately 0.4 V vs Ag|AgCl|Cl^–^, likely corresponded to the oxidation of the hydroxylated species obtained during hydrogen coevolution, whereas the peak centered at 0.1 V vs Ag|AgCl|Cl^–^ was attributed to the oxidation of a hydrogenated form of Ni. Before these two oxidation peaks, a small one appearing at approximately −0.25 V vs Ag|AgCl|Cl^–^ can be attributed to the oxidation of the more hydrogenated deposited Ni.^47−50,52,53^

Similar behavior was observed in the cathodic scan over the polycarbonate membrane coated with an Au layer (Figure 1b), although lower current densities were detected as well. The voltammetric profile also revealed that the current of platinum reduction was followed by the alloy’s deposition. Although, the onset of the Pt deposition process occurred at less negative potentials, the *j*/*E* slope of the Ni–Pt deposition was significantly lower than that of the Si/Ti/Au substrates. The sufficiently large size of the pore channels, at approximately 100 nm, and the gold seed layer facilitated the onset of the deposition, despite also lowering the deposition rate. In general, more negative potentials can be detrimental because the intense formation of hydrogen bubbles inhibits the growth of NWs. Thus, the data suggest that the most adequate potential range for the deposition of alloyed mesoporous Ni–Pt NWs was from −0.7 to −1.1 V vs Ag|AgCl|Cl^–^.

### Electrodeposition and Characterization of Mesoporous Films

**Figure 2:**
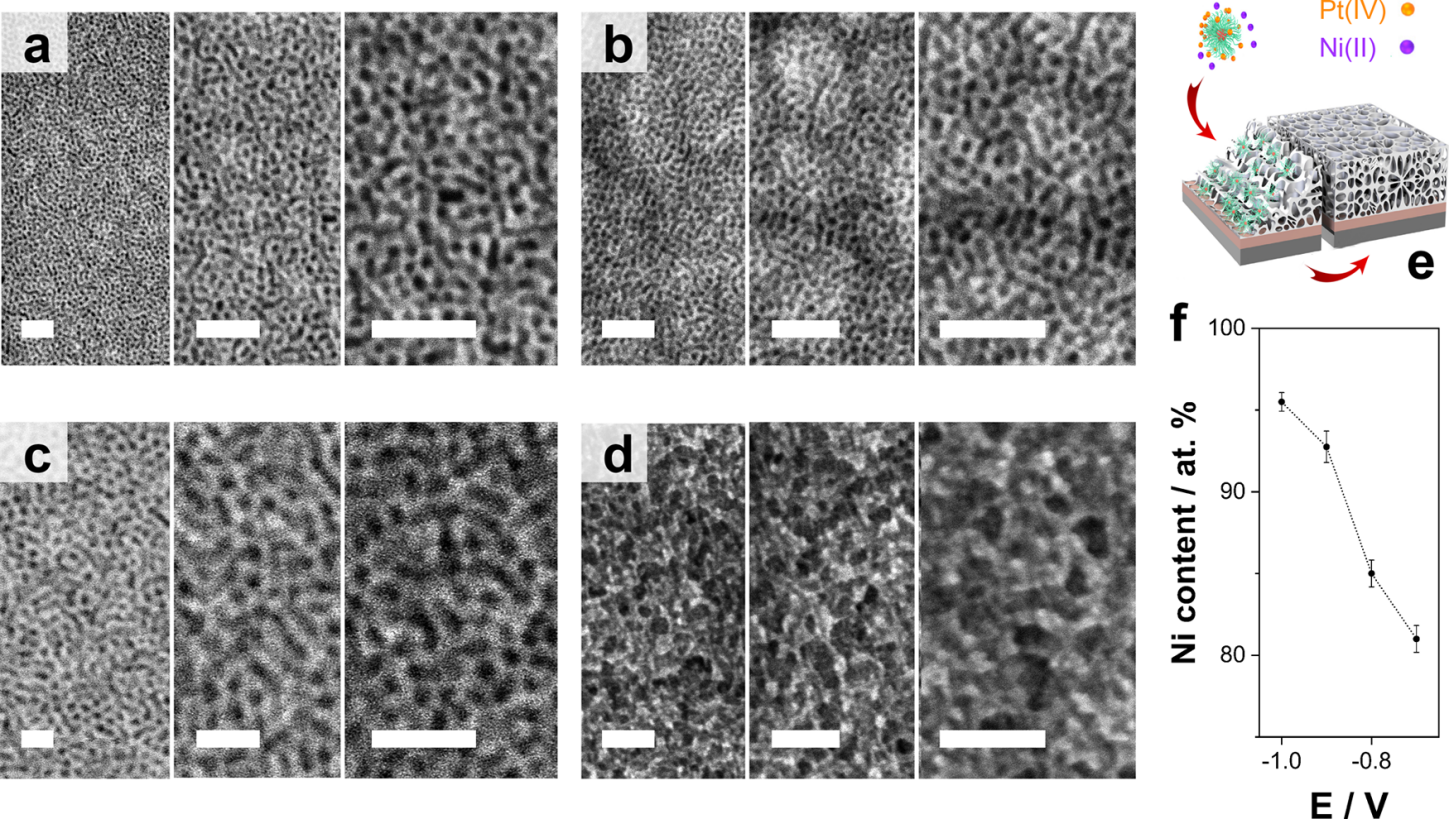
FE-SEM micrographs, at various magnifications, of mesoporous Ni–Pt films at (a) −0.7 V, (b) −0.8 V, (c) −0.9 V, and (d) −1.0 V vs Ag|AgCl|Cl^–^ after circulating at 0.45 C cm^–2^. Scale bar: 50 nm. (e) Schematic representation of the electrodeposition of mesoporous Ni–Pt films via block copolymer template electrodeposition. Adapted with permission from ref (54). (f) Ni content as a function of the electrodeposition potential used to electrosynthesize the mesoporous films.

As shown in Figure 2, well-defined, homogeneously distributed Ni–Pt mesoporous films with globular pores greater than 10 nm in size were potentiostatically electrodeposited at different potentials (charge density = 0.45 C cm^–2^). In this case, the mesoporous definition obtained closely related to the electrodeposition potential. Because Pt(IV) species interact with the hydrophilic portions of micelles, which themselves act as porogen agents in the soft-template system, applying negative potentials lowered the porosity of deposits while at once favoring Ni deposition (Figure 2c). This trend confirms the relevance of the interaction between the [PtCl_6_]^2–^ complex and the hydrophilic part block copolymer micelles.^50,53^ From there, a compromise between the Ni content and the integrity of mesopores was required to obtain well-defined Ni-rich Ni–Pt mesoporous deposits.

### Electrodeposition and Characterization of Mesoporous NWs

The electrodeposition of mesoporous NWs followed a double-template electrodeposition strategy based on the combination of two components: a hard-template polycarbonate membrane coated with an Au layer exhibiting a nominal pore diameter of 100 nm for defining the shape of NWs and a soft-template system (i.e., a block copolymer micellar aqueous solution) for defining the mesoporous architecture.^34,40,55,56^ Pt and Ni-rich Ni–Pt mesoporous were potentiostatically electrodeposited at −0.35, −0.80, and −1.00 V vs Ag|AgCl|Cl^–^ under stagnant conditions. The same deposition charge density (3.5 C cm^–2^) was employed for all of the samples.

**Figure 3:**
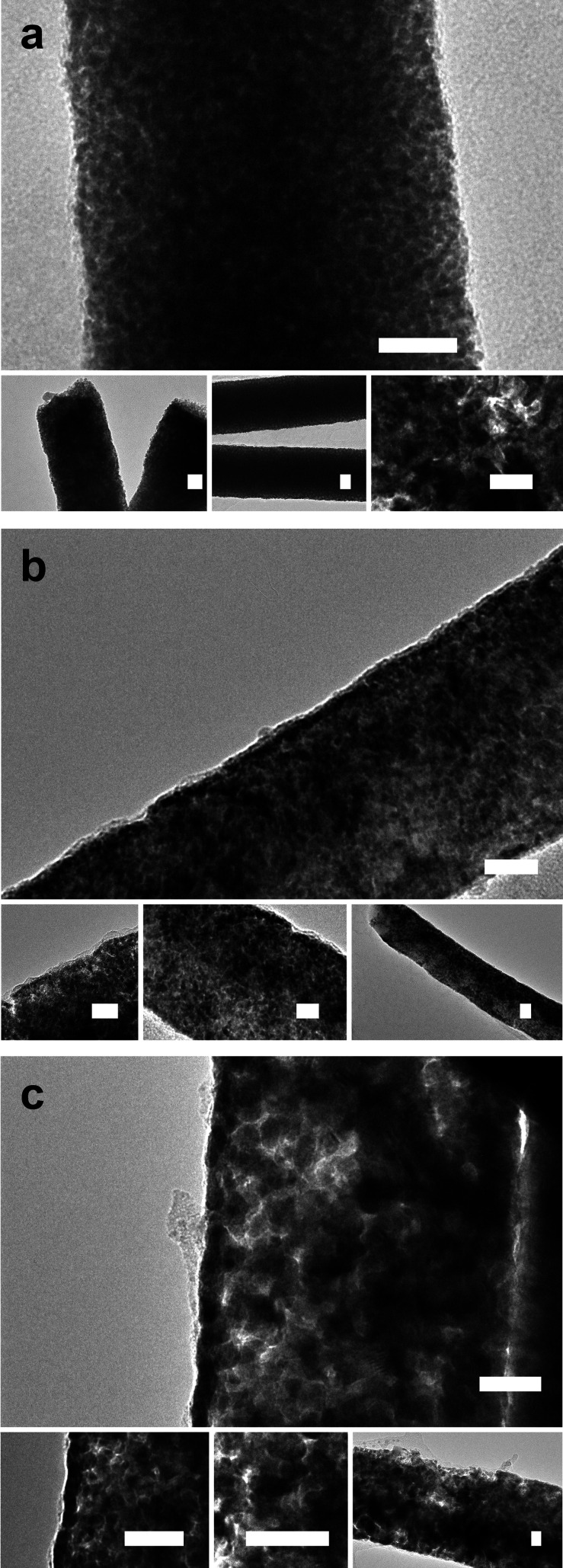
TEM micrographs, at various magnifications, of mesoporous NWs prepared at (a) −0.35 V, (b) −0.80 V, and (c) −1.0 V vs Ag|AgCl|Cl^–^ after circulating at 0.45 C cm^–2^. Scale bar: 20 nm.

As shown in Figure 3, straight, well-defined mesoporous Pt and Ni-rich Ni–Pt NWs were successfully deposited, which confirmed that the approach can generate mesoporous NWs with a high degree of superficial porosity and pore sizes ranging from 6 to 12 nm depending on the electrodeposition potential. As expected, for Ni–Pt mesoporous deposits, the pore definition was higher for the low deposition potentials at which alloy deposition occurs, conditions in which electrodeposition of Pt was most relevant. Table 1 summarizes the elemental compositions, dimensions, pore sizes, and BET surface areas of the obtained NWs.

**Table 1:** Potential, Nanowire Dimensions, Elemental Chemical Composition, and BET Surface Area of Electrodeposited NWs

potential (V vs Ag|AgCl|Cl^–^)	NW length (μm)	NW diameter (nm)	pore diameter (nm)	Ni (atom %)	BET surface area (m^2^ g^–1^)
–0.35	11.1 ± 0.6	∼110	4–6		160
–0.80	8.9 ± 0.8	∼108	4–7	78	155
–1.00	7.8 ± 0.7	∼110	5–11	94	138

The length of the NWs was consistent with the expected current efficiencies. At low deposition potentials, hydrogen coevolution was low, if not negligible, which resulted in high current efficiency, and larger NWs were consequently obtained after circulating the same charge density. However, the diameter of NWs, defined solely by the diameter of the channel of the polycarbonate membranes, did not correlate to the potential. Pore definition was also affected by the deposition potential because the pore diameter was slightly greater at more negative potentials, although the difference was not significant. The average current efficiencies, estimated by Faraday’s law of theoretical mass and the mass determined by ICP-OES, were 85, 70, and 60%, lower as negative applied potential was.

The elemental composition was primarily controlled by the deposition potential. A gradient of the composition was detected across the length of NWs, with a higher content of Pt at their extremes, possibly due to the proximity of the extremes to the Au substrate layer. However, the difference in the Pt content from one extreme to the other of NWs is negligible for NWs prepared at potentials below −0.9 V vs Ag|AgCl|Cl^–^ and lower than 8% for the NWs deposited at −1.0 V vs Ag|AgCl|Cl^–^. This effect has been previously described for the deposition of Ni-rich Ni–Pt mesoporous films on vitreous carbon.^50^ The oxygen content was negligible at −0.35 V vs Ag|AgCl|Cl^–^ but was more important at the more negative potentials. Therefore, the electrodeposition method from the selected bath and conditions allows the preparation of Ni-rich Ni–Pt mesoporous NWs with controlled composition.

**Figure 4:**
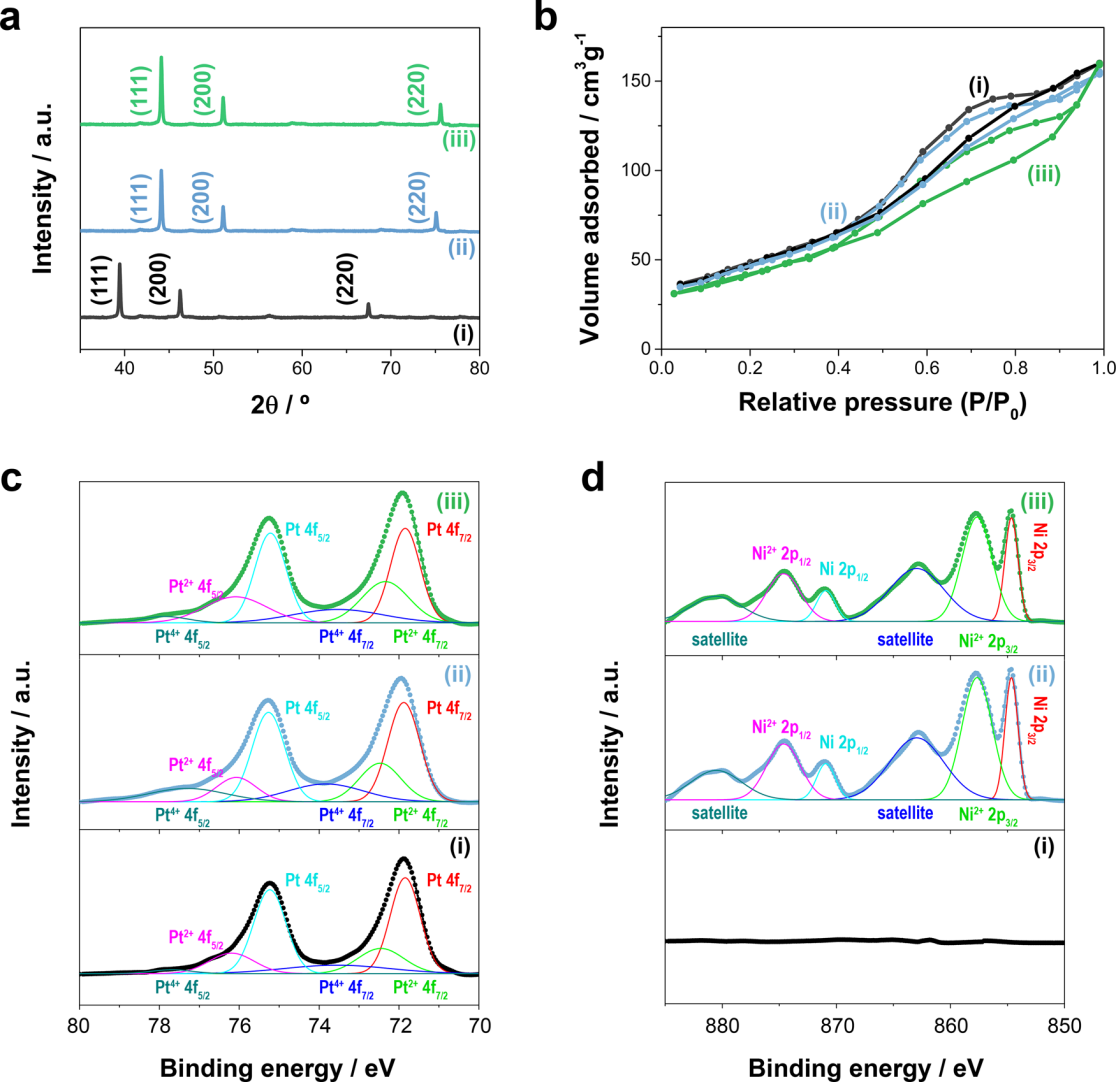
(a) XRD patterns, (b) nitrogen adsorption–desorption isotherms, and (c) Pt 4f and (d) Ni 2p XPS spectra of mesoporous NWs prepared at (i) −0.35 V, (ii) −0.80 V, and (ii) −1.0 V vs Ag|AgCl|Cl^–^ after circulating at 0.45 C cm^–2^.

Representative XRD patterns of Pt and both Ni-rich Ni–Pt mesoporous NWs are shown in Figure 4a. As expected, Ni-rich Ni–Pt mesoporous NWs can be indexed as a face-centered cubic Ni (111), (200), and (220) structure distorted by the incorporation of platinum. On the other hand, the pure Pt mesoporous NWs presented a face-centered cubic Pt (111), (200), and (220) structure. All of the NWs had a preferred orientation along the (111) direction, with small intensities at the (200) and (220) reflections. These results are consistent with the reported Ni-rich Ni–Pt mesoporous films.^50,57^

Nitrogen adsorption–desorption isotherms of mesoporous NWs prepared at (i) −0.35 V, (ii) −0.80 V, and (iii) −1.0 V vs Ag|AgCl|Cl^–^ are presented in Figure 4b, and showed BET surface areas of 160, 155, and 138 m^2^ g^–1^, respectively. These values are comparable to those of other mesoporous NWs reported elsewhere. The BET surface areas decreased as the Ni content in NWs increased, which is explained by the loss of the pore definition. However, high BET surface areas, significantly higher than those of compact NWs of 100 nm in diameter (i.e., <90 m^2^ g^–1^), were observed in all of the cases. The average pore size in the mesoporous NWs was approximately 3.8, 4.9, and 5.8 nm estimated by Barrett–Joyner–Halenda analysis for NWs prepared at (i) −0.35 V, (ii) −0.80 V, and (ii) −1.0 V vs Ag|AgCl|Cl^–^, respectively. This is also well supported by the diameters obtained from the TEM observation (Table 1).

XPS analyses were conducted to further investigate the chemical composition and chemical states of the elements in the prepared mesoporous NWs. All of the XPS spectra were corrected relative to the binding energy of the C 1 s peak (284.6 eV). As shown in Figure 4c, all of the samples exhibited the complex spectrum of Pt 4f, which confirmed the presence of Pt in all of the mesoporous NWs; the two characteristic peaks corresponding to Pt 4f_7/2_ and Pt 4f_5/2_ were observed.^58−61^ Note that each of these two peaks was fitted into three peaks. The most intense peaks correspond to metallic Pt 4f_7/2_ (∼71.9 eV) and Pt 4f_5/2_ (∼75.2 eV), whereas the weaker doublet at ∼72.8 and ∼76.4 eV and the weakest doublet at ∼73.6 and ∼77.6 eV were assigned to Pt(II) and Pt(IV), respectively.^58,60,61^ Note that the main chemical state of Pt in the surface of all of the mesoporous NWs was the metallic state. However, the Pt(II) and Pt(IV) contents increased slightly at more negative potentials. This trend is attributed to the key simultaneous hydrogen evolution, which is more relevant at increasingly negative preparation potentials. The proton consumption inside the nanochannels of the PC membranes translated into an increase of the local pH in the vicinity of the growing NWs, which likely promoted Pt(IV) deposition. However, zero-valence Pt atoms were found dominant based on the XPS analysis. The complex spectrum of Ni 2p is presented in Figure 4d. No assignable peaks were detected for the mesoporous NWs prepared at −0.35 V vs Ag|AgCl|Cl^–^, as expected for pure Pt mesoporous NWs. For the mesoporous NWs prepared at more negative potentials where Ni was also codeposited, the peaks corresponding to the nickel complex appeared at binding energies of ∼854.7 and ∼870.9 eV, ∼857.7 and ∼874.6 eV, and ∼862.9 and ∼880.5 eV, which correspond to the metallic Ni 2p_3/2_ and Ni 2p_1/2_, Ni(II) Ni 2p_3/2_ and Ni 2p_1/2_, and the nondepreciable satellite peaks, respectively.^58,62^ The surface atomic ratio of Ni(0) against Ni(II) was estimated to be 4:7 for NWs prepared at −0.80 and −1.0 V vs Ag|AgCl|Cl^–^. The significant deposition of Ni(II) species, possibly NiO and Ni(OH)_2_, may be attributed to the increase in the local pH close to the surface of the working electrode inside the nanochannel of the PC template. All of the findings are consistent with the results of the energy-dispersive spectrometry (EDS) analyses.

### Levulinic Acid Hydrogenation

Mesoporous NWs of Pt and Ni–Pt with different Ni contents were used to hydrogenate LA at 180 °C with FA as the hydrogen source. Selected results are summarized in Table 2. The conversion of LA and GVL product formation were not detected in the absence of the catalysts but observed only in the presence of Pt and Ni-rich Ni–Pt mesoporous NWs. The nearly complete conversion of LA (>99%) and a quantitative yield of GVL (>99%) were obtained at 180 °C after 180 min of the reaction. Concerning the total number of Pt and Pt–Ni atoms in the catalyst for the Ni-rich Ni–Pt mesoporous NWs at the selected conditions, TOFs (h^–1^) increased in the following order: Ni_94_Pt_6_ (38 h^–1^), Ni_78_Pt_22_ (50 h^–1^), and Pt (111 h^–1^). The highest value was observed for the Pt mesoporous NWs, although the activity of the Ni–Pt mesoporous NW was not negligible.

**Table 2:** Comparison of Catalytic Performances of Pt and Ni-Rich Ni–Pt Mesoporous NWs

catalyst	catalyst dose (mg)	Ni (atom %)	temperature (°C)	time (min)	conversion (%)	GVL selectivity (%)	TOF (h^–1^)
Pt	5		180	180	100	>99	111
Ni_78_Pt_22_	5	78	180	180	100	>99	50
				120	100	>99	76
				90	98	87	90
			140	180	100	>99	50
				120	100	>99	74
				90	90	70	63
			120	300	98	88	27
				180	87	68	31
				120	72	57	30
Ni_94_Pt_6_	5	94	180	180	100	>99	38
				120	94	82	30

**Figure 5:**
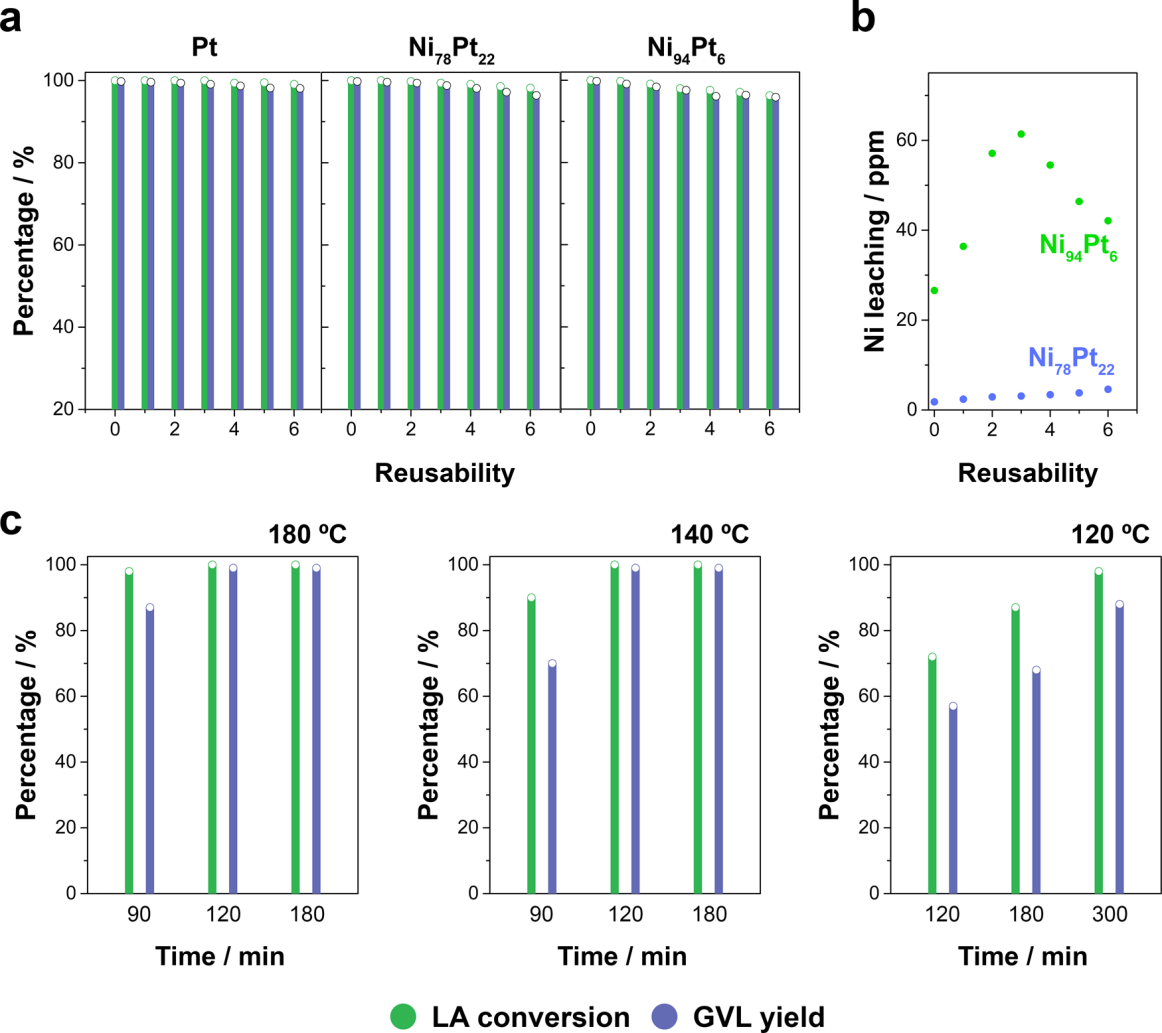
(a) Reusability experiments of the conversion of LA and FA into GVL over Pt and Ni-rich Ni–Pt mesoporous NWs (reaction conditions: 1 g of LA, catalyst loading = 5 mg, 180 °C, 3 h). (b) Nickel leaching of Ni_78_Pt_22_ and Ni_94_Pt_6_ catalysts after each reusability experiment (reaction conditions: 1 g of LA, catalyst loading = 5 mg, 180 °C, 3 h). (c) LA conversion and GVL yield over Ni_78_Pt_22_ mesoporous NWs (reaction conditions: 1 g of LA, catalyst loading = 5 mg).

The reusability of Ni-rich Ni–Pt mesoporous NWs during the hydrogenation of LA into GVL was investigated and compared with that of pure Pt mesoporous NWs (Figure 5a). The conversion of LA and the yield of GVL for the pure Pt catalysts remained constant during six consecutive recycling experiments, whereas the catalytic performance of the Ni-rich Ni–Pt mesoporous NWs decreased slightly after two and four recycling experiments for Ni_94_Pt_6_ and Ni_78_Pt_22_, respectively. However, both the LA conversion and GVL yield remained higher than 96% after the six successive runs, which indicates the superior stability of the Ni-rich Ni–Pt mesoporous NWs. After that, the catalysts were recovered from the reaction media and reused without any treatment, which indicates the low poisoning of both Pt and Ni–Pt mesoporous NWs during the reaction. The slight decrease in the catalytic performance of the Ni-rich Ni–Pt catalysts can be attributed to Ni leaching during the reaction, as consistent with what was observed for Ni_94_Pt_6_, in which the supernatant product was slightly green upon the reaction’s completion. For this reason, the level of Ni leaching was determined after each recycling experiment by inductively coupled plasma-optical emission spectrometry (ICP-OES) analysis. As shown in Figure 5b, Ni leaching was minimal for Ni_78_Pt_22_ but not negligible for Ni_94_Pt_6_. After the third rerun, the Ni leaching began to decrease for Ni_94_Pt_6_. In light of these results, Ni–Pt mesoporous NWs seem to be competitive catalysts for obtaining GVL and afford the full conversion (approx. 100%) of LA and quantitative yields of GVL (>99%) with negligible poisoning. The stability of Ni-rich Ni–Pt mesoporous NWs in working conditions is not excessively high when the Ni content exceeds 80 atom %; however, at percentages exceeding 75–80 atom %, their relatively high chemical stabilities make them ideal candidates for hydrogenating LA using FA. Such catalytic performance can be attributed to the synergetic effect of the Ni and Pt species and the high amount of the accessible surface area provided by the mesoporous surface and the architecture of NWs. Beyond that, the reduced consumption of noble Pt in these catalysts strongly supports their use vs the significantly more expensive Pt.

The effects of two critical parameters that significantly determine the energy consumption and global cost of GVL formation, that is, reaction time and temperature, were explored to confirm the outstanding catalytic performance of Ni_78_Pt_22_ mesoporous NWs. As shown in Figure 5c, Ni_78_Pt_22_ mesoporous NWs showed the complete conversion of LA (approx. 100%) and a quantitative yield of GVL (>99%) at 140 °C after 120 min. After 90 min at 140 °C or 180 °C, the conversion of LA was relatively high (>90%), although the GVL yield decreased to 70 and 87% at these respective temperatures. By contrast, at 120 °C, complete hydrogenation of LA was not observed after 300 min of reaction time. The optimal conditions of reaction time and temperature for achieving a quantitative production of GVL were thus close to 120 min and 140 °C, respectively. As shown in Table 3, Ni_78_Pt_22_ mesoporous NWs showed excellent catalytic performance in the hydrogenation of LA and GVL production. The catalytic performance was even possibly better than state-of-the-art heterogeneous catalysts for the hydrogenation of LA that use FA as a source of hydrogen.

**Table 3:** Hydrogenation of LA to GVL by Various Catalysts

catalyst	catalyst dose (g)	H_2_ source	temperature (°C)	time (min)	LA conversion (%)	GVL selectivity (%)	ref
Ni–Pt	0.005	formic acid	140	120	100	99	this work
Cu–Ni	0.125	H_2_ gas	150	180	95	70	(63)
Cu/Ni/Mg/Al	0.100	H_2_ gas	140	180	100	100	(64)
Ni/NiO	0.200	H_2_ gas	110	1440	100	>99	(65)
Ru/ZrO_2_	0.250	formic acid	150	720	73	73	(66)
Au–Ni(CI)/g-Al_2_O_3_(C)	0.600	formic acid	190	120	89	86	(66)
Ru/TiO_2_	0.050	H_2_ gas	200	240	100	98	(67)
Ru-P/SiO_2_		formic acid	170	720	96	>96	(23)
Ru/C		H_2_ gas	265	3000	100	99	(68)
Au–Pd	0.600	H_2_ gas	200	600	100	99	(69)
Ru/C		H_2_ gas	130		100	84	(70)
Cu/ZrO_2_	0.200	H_2_ gas	150	180	100	100	(71)
Ru	0.005	H_2_ gas	120	120	100	99.6	(72)
Fe–Re/TiO_2_	0.023	H_2_ gas	180	240	>98	95	(14)
Ru_1_/Fe_3_O_4_	0.060	H_2_ gas	150	120	99	99	(11)
CuAg	0.150	H_2_ gas	180	240	100	100	(73)
Ru-HAP	0.100	H_2_ gas	70	240	99	99	(74)
Ni–Cu/SiO_2_	1.000	formic acid	265	600	100	98	(75)
Ru	0.300	formic acid	190	300	81	57	(25)
Ru/Al_2_O_3_	0.200	H_2_ gas	130	30	100	99	(76)
Ni–MoO*_x_*	0.006	H_2_ gas	140	300	100	97	(77)
RuSn	0.500	H_2_ gas	180	240	100	99	(15)

^**1**^H NMR spectroscopy was performed to identify the species present during the reaction at different times. The ^**1**^H NMR spectra of LA (Figure 6a), dissolved in acetone-*d*_6_, showed two sets of signals: (i) two triplet resonances centered at 2.73 ppm (2H, *t*, ^3^*J* = 6.5 Hz, CH_3_COC**H**_2_−) and at 2.50 ppm (2H, *t*, ^3^*J* = 6.5 Hz, −C**H**_2_COOH) and (ii) a broad singlet peak at 2.12 ppm corresponding to the terminal methyl group. The multiplet centered at 2.05 ppm can be ascribed to the solvent. After 90 min of hydrogenation at 140 °C (Figure 6b), some residual LA signals remained visible because conversion had not been completed by the reaction time. Moreover, GVL signals appeared at (i) 4.61 ppm (1H, m) and (ii) 2.43, 1.82 ppm (4H, m), and 1.34 ppm (3H, d, ^3^*J* = 8.0 Hz, C**H**_3_−). Furthermore, the relationship of intensity between the LA and GVL signals indicated a high conversion due to the significantly higher intensity of the GVL signals than of signals ascribed to LA. After 120 min of the reaction at 140 °C, no LA signals were detected, thereby indicating its complete conversion into GVL (Figure 6c).

**Figure 6:**
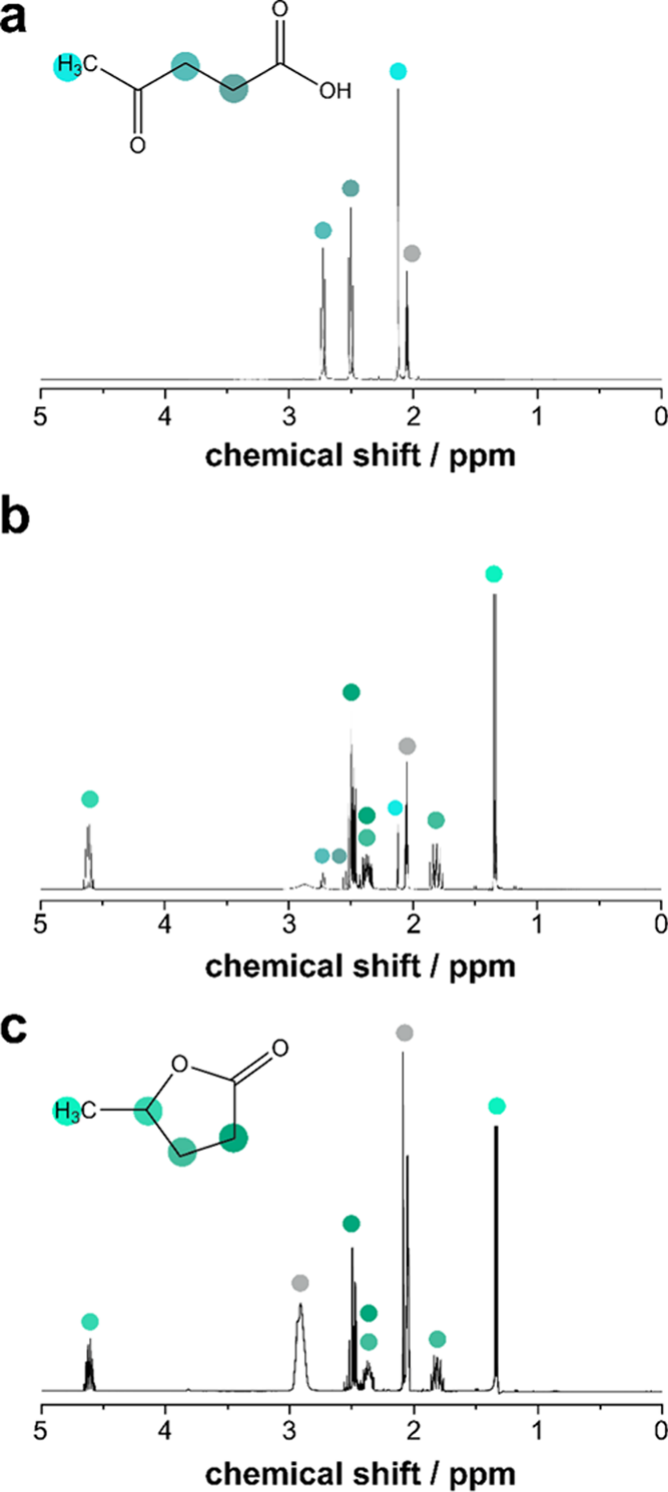
^1^H NMR spectra recorded in acetone-*d*_6_ of three reaction samples at (a) *t* = 0, (b) *t* = 90, and (c) *t* = 120 min of hydrogenation. Temperature = 140.0 °C. Dense gray circles correspond to the solvent signal.

## Conclusions

This work addressed two related objectives, the achievement of which required feedback between the intermediate results throughout the work. The preparation of effective catalysts was achieved using processes that required short times, comprised low noble metal contents, and achieved improved abilities for transforming biomass into profitable products under mild conditions. As with typical heterogeneous reaction systems, the increase of the surface–volume ratio was found to be one of the main issues, and the mesoporous morphology was determined as the factor for maximizing the ratio. Because the objective was a hydrogenation reaction, nickel was proposed as a partner to the efficient platinum catalyst, considering the well-established high efficacy and the lowest cost of nickel for hydrogenation.

To prepare the mesoporous nanostructures, the selected hard–soft-template-assisted electrodeposition has been demonstrated to be a very useful tool. The polycarbonate membrane confers shape control and the micellar electroactive solution imparts the mesoporous structure. Although it was demonstrated that the presence of [PtCl_6_]^2–^ in the electroactive solution is necessary as the main porogen inducer, its solution content reduced progressively and was replaced by the electroactive Ni ion species.

Platinum chloride species, due to their intrinsic negative charge, are the main species that interact with the micelles present in the solution. The micelles were promoted by the presence of the copolymer, which was mainly charged positively. The difference between the deposition potential of the electroactive species combined with the inert behavior of both nickel and platinum forces the application of strongly negative potentials during deposition. These differences correspond to high overpotentials for platinum deposition, which favors the deposition as the seed layer of the mesoporous nanostructure that is replicated during the alloy deposition.

In the selected solution, the increase in the negative applied potential promoted nickel deposition; however, it simultaneously enhanced the hydrogen coevolution, and the gas bubbles adversely affected the mesoporous morphology and caused metal corrosion. The strongest conditions led to the deposition of the nickel content at around 95%. However, the low platinum content is not sufficient for preventing the facile superficial corrosion of nickel that would carry the nickel species into the reaction medium, as has been demonstrated by the reusability experiments. Therefore, balancing of the applied potential was considered for ensuring the mesoporous character, chemical stability, and economic cost. Ni_78_Pt_22_ was the most suitable material that was obtained from the range of conditions analyzed for the preparation of the catalysts that exhibited pore diameters between 4 and 7 nm.

All of the prepared Ni-rich Ni–Pt catalysts promoted the complete conversion of levulinic acid to γ-valerolactone, albeit under longer reaction times in comparison to other similar conditions, than those achieved with free-Ni–Pt. For all of the catalysts prepared, the temperature and time required for the complete conversion were analyzed. In all of the experimental conditions, a similar behavior was observed; when the temperature was lowered, the time required for the near-complete conversion increased. For the Ni_78_Pt_22_ catalyst, the best conditions were established as 140 °C and 120 min to achieve full conversion. Lowering the temperature by 20 °C required a nearly threefold increase in the reaction time.
